# Empowering Mental Health Inpatients: Insights Into Voting Rights Awareness and Support

**DOI:** 10.1192/bjo.2025.10297

**Published:** 2025-06-20

**Authors:** Nahid Patel, Elizabeth Keeper, Mark Winchester

**Affiliations:** 1 University Hospital North Midlands, Stoke on Trent, United Kingdom; 2 Midlands Partnership University Foundation Trust, Stafford, United Kingdom

## Abstract

**Aims:** This study explores the knowledge, barriers, and support related to voting rights among mental health inpatients and staff at an inpatient mental health hospital. It evaluates the impact of an educational intervention implemented prior to the July 2024 UK General Election, aimed at enhancing staff knowledge and supporting patient participation in the electoral process.

The aim of this study is to evaluate staff and patients’ views on inpatient voting rights, identify barriers to participation, and evaluate the impact of an educational intervention on staff knowledge and patient support.

**Methods:** Surveys completed by 92 staff members pre-intervention and 28 staff members post-intervention to assess knowledge of voting eligibility, barriers to participation, and support strategies. Patient surveys were completed by 53 patients pre-election and 37 post-election, exploring their awareness, voting intentions, and challenges.

**Results:** Post-intervention, staff knowledge significantly improved, with 75% correctly identifying the voting rights of patients without capacity, up from 36%. Additionally, 17.6% of staff correctly identified all voting eligibility categories pre-intervention, with notable improvement afterwards. Common barriers reported by staff included lack of awareness, legal uncertainties, and logistical challenges. Among patients, 85% believed they had the right to vote, but only 43% intended to vote. Post-election, 81% of patients were aware of the general election, yet only 14% participated in voting. Barriers to participation included physical challenges and voter registration issues, with limited support contributing to low turnout.

**Conclusion:** The educational intervention enhanced staff knowledge of voting rights, but significant barriers remain, particularly around patient registration and logistical support. Despite high awareness among patients, low turnout highlights the need for continued staff training and efforts to address barriers. The findings from this research will guide the development of a trust-wide policy to support inpatient voting in future elections.

